# Complimentary effect of yogic sound resonance relaxation technique in patients with common neck pain

**DOI:** 10.4103/0973-6131.66774

**Published:** 2010

**Authors:** Bali Yogitha, R Nagarathna, Ebnezar John, HR Nagendra

**Affiliations:** Department of Orthopaedics, Ebnezar Orthopedic Centre, Parimala Hospital, Bengaluru, India

**Keywords:** Neck pain, mind sound resonance technique, physiotherapy, stress, yoga

## Abstract

**Background:**

Studies have shown that conventional treatment methods with drugs, physiotherapy and exercises for common neck pain (CNP) may be inadequate. Yoga techniques have been found to be effective complimentary therapies in chronic low back pain and also for stress reduction in other diseases.

**Objective:**

The aim of the study was to examine the complimentary role of a yogic relaxation called mind sound resonance technique (MSRT) in non-surgical management of CNP.

**Materials and Methods:**

In this randomized controlled study, 60 patients with CNP were assigned to two groups (yoga, *n*=30) and (control, *n*=30). The yoga group received yogic MSRT for 20 minutes in supine position after the conventional physiotherapy program for 30 minutes using pre-recorded audio CD and the control group had non-guided supine rest for 20 minutes (after physiotherapy), for 10 days. MSRT provides deep relaxation for both mind and body by introspective experience of the sound resonance in the whole body while repeating the syllables A, U, M and Om and a long chant (*Mahamrityunjaya mantra*) several times in a meaningful sequence. Both the groups had pre and post assessments using visual pain analog scale, tenderness scoring key, neck disability score (NDS) questionnaire, goniometric measurement of cervical spinal flexibility, and state and trait anxiety inventory-Y1 (STAI-Y1).

**Results:**

Mann-Whitney U test showed significant difference between groups in pain (*P*<0.01), tenderness (*P*<0.01), neck movements (*P*<0.01). NDS (*P*<0.01) and state anxiety (STAI-Y1) showed higher reduction in yoga (*P*<0.01) than that in the control group. Wilcoxon’s test showed a significant improvement in both groups on all variables (*P*<0.01).

**Conclusions:**

Yoga relaxation through MSRT adds significant complimentary benefits to conventional physiotherapy for CNP by reducing pain, tenderness, disability and state anxiety and providing improved flexibility.

## INTRODUCTION

Neck pain is one of the very common complaints across the globe, with a prevalence of nearly 13%[1,2] and a lifetime prevalence of nearly 50% and women are more prone than men with an incidence ratio of 1.67 (women are more likely than men to develop neck pain; incidence rate ratio=1.67, 95% confidence interval 1.08-2.60).[3–5] Neck and shoulder pain has also become an increasingly common health complaint among adolescents, where the prevalence is found to be higher in girls than in boys.[6] It is one of the frequent causes for sickness absenteeism that could disrupt a nation’s economy apart from disrupting the personal and professional life of a victim.[7] Though the exact cause is unknown, altered neck mechanics, advanced age-related changes, additional load on the neck, occupational hazards as in computer professionals or call center workers, faulty sleeping habits and sudden violent jerking injuries to the neck as in whiplash injury are some of the etiological factors.[8] “Common neck pain” (CNP) which is not due to any organic lesion accounts for more than 80% of neck pains.[9] Psychological stress that may be associated in any of these factors cannot be undermined.[10] Depression and anxiety are well-known undesirable side effects of chronic neck pain.[11,12]

Since the underlying pathology of neck disorders remains unclear, the treatments are aimed at relief of pain and stiffness. The conventional conservative methods include non-steroidal anti-inflammatory drugs, physical measures such as heat, ultrasound, manipulation and exercises.[13]

Moffett *et al*, compared a brief physiotherapy intervention on 268 patients (for 7 days) with usual physiotherapy (for 14 days) for CNP and showed that latter may be only marginally better than the former.[14]

Spray and stretch (vapo-coolant spray followed by passive stretching) was compared to laser therapy and a placebo, with no significant difference between the groups and no significant reduction in pain.[15] A study conducted to investigate the use of traction in two randomized controlled trials revealed the difference between the groups to be small and not significant.[16,17] Loy *et al*, showed that symptomatic improvement was better with a combination of cervical traction, short wave diathermy and electro acupuncture, than a combination of TENS, collar, rest and education in moderate quality neck pain.[18] With growing dissatisfaction with these conventional therapies, there is a pressing need for complementary measures and yoga seems to hold promise through its multifaceted approach to healing. Studies have established the role of yoga in decreasing the pain and disability in chronic low back pain, along with improved flexibility within 1 week to 4 months of yogic intervention with no adverse effects.[19]

Yoga has also been found to be an effective tool in reducing stress levels.[20,21] Mind sound resonance technique (MSRT) is one of the advanced guided yoga relaxation techniques that can be practiced in supine or sitting posture for achieving the goal of positive health, will power, concentration and deep relaxation.

This tool [Table 1] was developed using the concepts from traditional texts that talk about the power of Om (*Mandukya Upanishad*) and *Nadanusandhana* (*Hatha Yoga Pradipika*) for achieving internal mastery over the modifications of the mind (Patanjali’s definition of yoga).[22] MSRT opens up the secret of traditional chants called *Mantras*. MSRT was one of the components of the intensive integrated yoga program that was used as the intervention for low back pain study.[23] Although MSRT has been used routinely as a component of the integrated approach to yoga therapy for treatment of neck pain and back pain at our yoga therapy health home and the orthopedic center with encouraging results, the results of these studies were not published. Hence, this study was planned with an aim to evaluate the efficacy of an add-on program of this yoga-based relaxation technique and compare it with the conventional physiotherapy technique. The hypothesis was that the yoga group would show better improvement than the control group in measures of pain, tenderness, disability, flexibility and state anxiety.

**Table 1:** Steps of MSRT

Practice	Duration
Prayer – salutation to the divine (*Maha Mrityunjaya Mantra*)	1 minute
Quick relaxation technique – observe the abdominal breathing internally with closed eyes	3 minutes
Loud chanting (*Ahata*) of A, U, M and AUM (three rounds)	16 minutes
Alternate loud (*Ahata*) and mental (*Anahata*) chanting of A, U, M and AUM (three rounds)	
*Ahata* of a long chant invoking fearlessness – *Maha Mrityunjaya Mantra* (three rounds)	
Alternate *Ahata–anahata* of *Mahamrityunjaya* mantra (three rounds)	
*Anahata* of AUM (three rounds)	
Silence	
Resolve	
Closing prayer for peace	

## MATERIALS AND METHODS

The sample size was derived by calculating the effect size based on the mean and standard deviation (SD) of an earlier unpublished interventional study done at this center using the same design for chronic low back pain, by Anupritha *et al*[23] Eighty-seven consecutive patients who came to the Ebenezer’s orthopedic unit of Parimala hospital, Bengaluru, India, for treatment of neck pain were screened. Of these, 60 who needed physiotherapy and consented to be in the study were randomized into two groups of 30 each using a computer-generated random number table on the “randomizer.com” software. There were 28 females and 32 males.

The institutional ethical committee of SVYASA approved the study. Signed informed consent was obtained from all the participants.

Patients with CNP due to spasm (myalgia) or strain of the neck muscles, ligament strain, cervical spondylosis without any neurological impairment and who were advised physiotherapy by the consulting orthopedic surgeon were included in the study. It was ensured that these were literate patients in the age group of 20–70 years with no previous exposure to yoga.

Those with uncommon neck pains (UCNP) due to organic causes such as congenital conditions like wry neck, infective conditions like tuberculosis, inflammatory conditions like rheumatoid arthritis, metabolic disorders like osteoporosis, neoplastic conditions and post-traumatic conditions with ligament or bone injuries were excluded.

The study design was as follows. This was a randomized parallel two-armed control design. Sixty subjects who were advised conventional treatment including physiotherapy for CNP at the orthopedic centre were selected for the study and were randomized into two groups after obtaining the informed consent. Yoga group received yoga-based relaxation technique that included MSRT after a short quick relaxation technique, by way of a prerecorded audio tape played with head phones for a period of 20 minutes, after 30 minutes of conventional physiotherapy. Control group had non-guided supine rest for 20 minutes after the conventional physiotherapy. After randomization, the pre-data on all variables were recorded. The role of stress and the value of relaxation in general after the conventional physiotherapy were explained to both the groups by the research officer. The yoga group had a separate session to explain the meaning and other details of the intervention and was taught the technique through personal instructions by the yoga therapist for half an hour on the 1^st^ day. From the second day onward, they were asked to practice the same in supine position by listening to the prerecorded audio tape on head phones in the annex room of the physiotherapy department of the hospital.

The subjects in the control group were asked to relax comfortably and calm down their mind in the supine rest on their own in the annex room similar to the study group. Post data were obtained on all subjects on the 10th day.

As this was an interventional study, there was no possibility of blinding. The pain analog scale (PAS) sheets and the answer sheets of State Trait Anxiety Inventory (Form1) (STAI Y1) were kept aside for data extraction until the completion of both pre and post data.

Assessments through the clinical examination by the orthopedic surgeon before recruitment included (a) history of all health problems followed by examination for assessment of the degree and type of neck pain, (b) neurological examination to look for any motor or sensory deficit, (c) X-rays of the cervical spine in antero-posterior and lateral views.

The primary outcome measures used were visual pain analog scale (PAS), neck muscle tenderness, neck disability score (NDS) and movements of the neck. The subjects were asked to mark the degree of their present pain on a numerical PAS by placing a dot on a 10-cm line drawn on a white paper with centimeter markings, with 0 = “nil pain” and 10 = “the worst possible pain the person can imagine”.[24] Neck muscle tenderness grading of tenderness was done using the following key: Grade 1 = tenderness on deep palpation of para-cervical muscles, Grade 2 = patient winces on pressure, Grade 3 = patient winces and withdraws and Grade 4 = patient does not allow one to touch.[25] The NDS developed by Vernon *et al*., was used.[26] It consists of 60 questions related to pain intensity, personal care, work, concentration, lifting, reading, driving, recreation, headache and sleeping. The patients were asked to complete the answers to these questions on a 6-point scale ranging from 0 to 5. Cervical spinal flexibility was measured by using a Lenthon Goniometer for the following movements of the neck: flexion (F), extension (E), lateral flexion (to right = LFR and to left = LFL), and lateral rotation (LRR and LRL).

Secondary outcome measures included blood pressure (BP), pulse rate (PR) and state anxiety inventory (STAI-Y1). BP was measured using a sphygmomanometer on day 1 and day 10 after the treatment. PR was counted manually for 1 minute before the treatment was started on 1st and 10th day.

STAI developed by Spielberger *et al* (1970) consists of two forms (Y1 and Y2) each comprising 20 items rated on a 4-point scale. and was used for assessing the anxiety levels. Form Y1 used to assess state anxiety is defined as “a transitory emotional state that varies in intensity, fluctuates over time and is characterized by feelings of tension and apprehension and by heightened activity of the autonomic nervous system”. It evaluates how respondents feel “right now” at this moment. Form Y2 evaluates trait anxiety, which is defined as “a relatively stable individual predisposition to respond to situations perceived as threatening”. It assesses how the respondents feel most of the time. The scores for each of the forms range from 20 to 80, with high scores indicating presence of high levels of anxiety. We used Y1 in our study.

Data sheets marked by all patients for PAS, NDS and STAI-Y1 were coded and kept aside for future assessment. All measurements were taken before the intervention on 1^st^ day and 10^th^ day.

### Intervention

Conventional schedule of physiotherapy that was common to both the groups included (a) intermittent cervical traction treatment (one-sixth of the body weight) for 10 minutes, using the Cervical Traction instrument, Electrocare (2001), Chennai, India (b) interferential therapy for 10 minutes using IFT Technomed (2003) and (c) ultrasound massage for 10 minutes using Ultrasound Technomed 408 (2003).

An add-on intervention for the control group was a non-guided supine rest for a period of 20 minutes after the conservative treatment (physiotherapy) for 30 minutes. Add-on yoga relaxation for the study group was used. After the physiotherapy, the study group received the yoga relaxation therapy called MSRT done in supine position. MSRT is one of the advanced yoga techniques for achieving deep relaxation. MSRT involves experiencing with closed eyes the internal vibrations and resonance developed while chanting the syllables A, U, M, Om and *Mahamrityunjaya mantra* sounds.

Instructions were given in the recorded tape to feel the resonance all over the body both during loud (*Ahata*: heard) and mental chanting (*Anahata*: unheard). This is done alternately starting from *Ahata* ‘A’ followed by *Anahata* ‘A’ repeated three times. This is followed by similar repetitions of all other chants. Resonance generated by MSRT helps in revitalizing the internal energy in the body. It takes to deeper layers of silence, wards off all fears and stresses. It can lead to an experience of tremendous expansion and rest that forms the basis of the healing power of these traditional chanting called *Mantras*.[27] This type of mindfulness techniques that involve deep levels of mind and body relaxation have the ability to reduce the sympathetic nervous system activation and increase parasympathetic nervous system activity and restore homeostasis.

### Data extraction

#### Pain analog scale

The distance of the point marked by the patient on the PAS line was measured by using a measuring scale and expressed in centimeters.

#### Spinal flexibility

The values for F, E, RLF, LLF, RLR and LLR were expressed in degrees.

#### Neck disability score

The total score was obtained by taking the sum of the scores for all 60 questions.

### State and trait anxiety inventory-Y1

The scoring of the STAI-Y1 was carried out as per the manual. The sum of the scores on the 5-point scale for the 12 questions marked on the answer sheets was considered as the total score for state anxiety.

### Data analysis

Data were analyzed using statistical package for social sciences (SPSS, version 10.0). The base line values of the two groups were checked for normal distribution by using Shapiro-Wilk’s Test. Since the parameters were not normally distributed, non-parametric tests were used. Wilcoxon’s signed ranks test was done to compare the means before and after intervention. The differences between the two groups for all variables were assessed by Mann-Whitney U test.

### Ethics

Ethical clearance was obtained from the ethical committee.

## RESULTS

**Figure 1:**
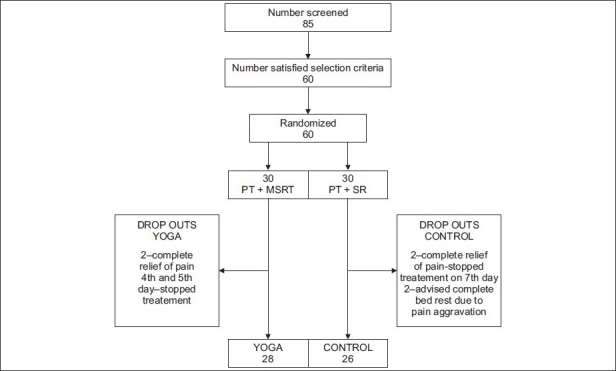
Trial profile

[Table 2 shows results of both between and within groups] Sixty subjects who satisfied the selection criteria were registered for the study of which 32 (15 in control, 17 in yoga) were females and 28 (15 in control, 13 in yoga) were males. Table 3 shows the baseline characteristics which were similar between groups. There were six dropouts (two in yoga and four in control group). The reasons for dropping out are mentioned in trial profile [Figure 1]. The mean and SD of age in yoga group was 41.03 ± 15.54 and that of control group was 42.23 ± 14.30 years. Duration of neck pain was 6.8 + 3.16 and 5.40 + 2.66 years for control group and yoga group, respectively. There was no significant difference between groups for baseline values on any of the variables. Table 4 shows the results within the groups after 10th day of the intervention.

**Table 2:** Table of results

Variables	Yoga group (%)			Control group (%)			Effect size
	Pre (M ± SD)	Post (M ± SD)	% change	Pre (M ± SD)	Post (M ± SD)	% change	
PAS	8.27 ± 1.14	0.37 ± 0.67	95.5*+	7.93 ± 1.14	3.07 ± 1.98	61.29*	1.83
Tenderness	2.37 ± 0.89	0.17 ± 0.38	92.82*+	2.23 ± 0.68	0.83 ± 0.65	62.78*	1.24
NDS	45.30 ± 21.49	3.93 ± 5.36	91.32*+	43.47 ± 19.82	13.90 ± 10.03	68.02*	1.24
Flexion	10.13 ± 7.94	44.60 ± 7.12	–340.27*+	7.67 ± 5.93	29.93 ± 5.42	–290.22*	2.32
Extension	8.40 ± 7.37	44.73 ± 7.16	–432.5*+	7.40 ± 5.51	29.10 ± 6.74	–293.24*	2.25
RLF	7.73 ± 4.92	37.23 ± 5.29	–381.63*+	7.30 ± 6.10	30.67 ± 5.49	–320.13*	0.94
LLF	8.13 ± 4.95	38.33 ± 5.20	–371.46*+	6.70 ± 5.93	30.90 ± 4.99	–361.19 (*)	1.46
RLR	8.60 ± 5.83	45.37 ± 7.58	–427.55*+	9.07 ± 5.51	29.87 ± 7.42	–229.32*	2.07
LLR	8.77 ± 5.07	44.13 ± 6.74	–403.19*+	10.30 ± 6.35	29.87 ± 7.16	–190*	1.9
STAI	56.80 ± 8.10	45.83 ± 10.66	19.31*+	58.13 ± 9.32	53.37 ± 5.64	8.18	0.88
BPS	132.30 ± 12.31	111.60 ± 9.31	15.64*+	134.53 ± 14.29	127.13 ± 15.28	5.50*	1.23
BPD	86.50 ± 8.12	72.93 ± 6.80	15.68*+	83.60 ± 16.62	83.30 ± 8.18	0.35	1.38
Pulse	75.30 ± 6.59	67.70 ± 5.54	10.09*+	76.23 ± 6.25	74.13 ± 6.66	2.75	1.05

* *P*<0.01 for Wilcoxon’s test (within groups);

+ *P*<0.01 for Mann-Whitney U test (between groups);

M = Mean, SD = Standard deviation, percentage and EF = Effect size of yoga and control groups, PAS = Pain analog scale, NDS = Neck disability score, RLF = Right lateral flexion, LLF = Left lateral flexion, RLR = Right lateral rotation, LLR = Left lateral rotation, STAI = State trait anxiety inventory, BPS = Blood pressure systolic, BPD = Blood pressure diastolic

**Table 3:** Demographic data

Characteristics	Yoga (n=30)	Control (n=30)
Age (M ± SD)	41.03 ± 15.54	42.23 ± 14.30
Gender		
Males	17	15
Females	13	15
Causes		
Non-specific	14	13
Spondylosis	16	17
Height	157.45 ± 7.40	158.35 ± 5.97
Weight	60.37 ± 11.07	59.23 ± 13.16
BMI	24.60 ± 4.15	23.90 ± 4.51

**Table 4:** Results after intervention

Variable	Yoga		Control	
	Pre (M ± SD)	Pre (M ± SD)	Pre (M ± SD)	Post (M ± SD)
PAS	8.27 ± 1.14	0.37 ± 0.67*	7.93 ± 1.14	3.07 ± 1.98*
TN	2.37 ± 0.89	0.17 ± 0.38*	2.23 ± 0.68	0.83 ± 0.65*
F	10.13 ± 7.94	44.60 ± 7.12*	7.67 ± 5.93	29.93 ± 5.42*
E	8.40 ± 7.37	44.73 ± 7.16*	7.40 ± 5.51	29.10 ± 6.74*
RLF	7.73 ± 4.92	37.23 ± 5.29*	7.30 ± 6.10	30.67 ± 5.49*
LLF	8.13 ± 4.95	38.33 ± 5.20*	6.70 ± 5.93	30.90 ± 4.99*
RLR	8.60 ± 5.83	45.37 ± 7.58*	9.07 ± 5.51	29.87 ± 7.42*
LLR	8.77 ± 5.07	44.13 ± 6.74*	10.30 ± 6.35	29.87 ± 7.16*
NDS	45.30 ± 21.49	3.93 ± 5.36*	43.47 ± 19.82	13.90 ± 10.03*
STAI-Y1	56.80 ± 8.10	45.83 ± 10.66*	58.13 ± 9.32	53.37 ± 5.64
BPBS	132.30 ± 12.31	111.60 ± 9.31*	134.53 ± 14.29	127.13 ± 15.28*
BPBD	86.50 ± 8.12	72.93 ± 6.80*	83.60 ± 16.62	83.30 ± 8.18
PB	75.30 ± 6.59	67.70 ± 5.54*	76.23 ± 6.25	74.13 ± 6.66

* *P* < 0.01 for Wilcoxon’s test (within groups)

**Table:** ABBREVIATIONS

PAS1	Pain analog scale 1st day	NDS10	Neck disability score 10th day
PAS10	Pain analog scale 10th day	STAI1	State trait anxiety 1st day
TN1	Tenderness 1st day	STAI10	State trait anxiety 10th day
TN10	Tenderness 10th day		
F1	Flexion 1st day	BPB1	Blood pressure 1st day, before intervention
F10	Flexion 0th day	BPB2	Blood pressure 1st day, during intervention
E1	Extension 1st day	BPB3	Blood pressure 1st day, after intervention
E10	Extension 10th day	BPA1	Blood pressure 1st day, before intervention
RLF1	Right lateral flexion, 1st day	BPA2	Blood pressure 1st day, during intervention
RLF10	Right lateral flexion, 10th day	BPA3	Blood pressure 1st day, after intervention
LLF1	Left lateral flexion, 1st day		
LLF10	Left lateral flexion, 10th day	PB 1	Pulse rate 1st day, before intervention
RLR1	Right lateral rotation, 1st day	PB 2	Pulse rate 1st day, during intervention
RLR10	Right lateral rotation, 10th day	PB3	Pulse rate 1st day, after intervention
LLR1	Left lateral rotation 1st day	PA1	Pulse rate 10th day, before intervention
LLR10	Left lateral rotation 10th day	PA2	Pulse rate 10th day, during intervention
NDS1	Neck disability score 1st day	PA3	Pulse rate 10th day, after intervention

Non-parametric Wilcoxon’s test showed a significant improvement in both the groups in pain (*P*<0.01), tenderness (*P*<0.01), NDS (*P*<0.01), spinal flexibility including flexion (*P*<0.01), extension (*P*<0.01), RLF (*P*<0.01), LLF (*P*<0.01), RLR (*P*<0.01) and LLR (*P*<0.01) movements of the neck and state anxiety (*P*<0.01). There were significant (*P*<0.05) differences between groups on all these variables studied, with higher percentage changes in yoga than control group. Systolic BP showed significant reduction in both the groups (*P*<0.01) but the diastolic BP and the PR showed significant reduction only in yoga group (*P*<0.01) with non-significant difference between groups.

In yoga group there was reduction in pain by 95.5%, tenderness by 92.82% and NDS by 91.32%. The spinal flexibility increased in movements of flexion by –340.27%, extension by –432.5%, RLF by –381.63%, LLF by 371.46%, RLR by –427.55%, and LLR by –403.19%.

In conclusion, it is observed that there is significant improvement in all variables in both the groups with significantly better improvement in yoga than control group.

## DISCUSSION

This prospective randomized control study was designed to assess the efficacy of addition of a yoga-based relaxation technique called MSRT to the conventional physiotherapy program for 10 days in patients with CNP. Analysis of outcomes indicated significant difference between the groups (Mann-Whitney test) and within groups (Wilcoxon’s test) for all variables including PAS (*P*<0.01), tenderness (*P*<0.01), flexion (*P*<0.01), extension (P<0.01), RLF (*P*<0.01), LLF (*P*<0.01), RLR (*P*<0.01), LLR (*P*<0.01), NDS (*P*<0.01) and state anxiety (STAI-Y1) of state and trait anxiety inventory (*P*<0.01).

Meaning and comparison of a few earlier studies suggest the usefulness of relaxation techniques in reduction of pain and improvement of flexibility by reduction in muscle tension in patients with chronic neck pain. Kabat–Zinn showed that 65% of the patients felt lesser pain after practicing mindfulness meditation for 10 weeks in patients with chronic pain, who had not improved with traditional medical care.[28] There are three randomized trial controls on yoga for chronic low back pain. RCTs using Viniyoga and Iyengar yoga therapy showed reduction in pain and functional disability with non-significant changes in the control group. In a study done on patients with chronic low back pain by Tekur *et al*, a short-term intensive residential yoga program was compared with intensive residential physical exercise program.

The yoga group showed significantly better improvement in pain-related disability and spinal flexibility.[19] There is no study that has used MSRT for chronic pain. One unpublished study at this institution (dissertation for MSc degree of Shetty A., 2006) on the role of MSRT in chronic low back pain showed reduction in back pain, improvement in spine flexibility and decrease in stress on using this relaxation technique. Sripada Swamy and Vasudha in a dissertation for M.Sc., Yoga, on Nādānusandāna have compiled information on the practice of nādānusandhāna, benefits and its application from ancient Indian scriptures as well as from the experts in the field of yoga and spiritual lore.[29]

A review on the evidence for mind body therapies such as guided relaxation, meditation, imagery and cognitive-behavioral therapy in the treatment of pain-related medical conditions concluded that these strategies may be an appropriate adjunctive treatment for chronic neck and low back pain as they offer better stress management techniques, coping skills training and cognitive restructuring.[30]

As for the mechanism, a research conducted by Linton, to review the psychological risk factors in back and neck pain indicated a clear link between psychological variables with neck and back pain. Results of the prospective studies showed that the psychological variables were related to onset of pain, acute, subacute and chronic pain. Stress, distress or anxiety as well as mood and emotions, cognitive functioning and pain behavior were found to be significant factors.[31]

As quoted in one study, tension that is associated with stress is stored mainly in the neck muscles, diaphragm and the nervous system. If these areas are relaxed, stress gets reduced, minimizing the impact of stress on the individual. It has also been suggested that the presence of depressive symptoms predicts future musculoskeletal disorders but not vice versa.[19] Stress can cause spasms by interfering with co-ordination of different muscle groups involved in the functioning of the neck.

Yoga is an ancient Indian science and way of life which includes the practice of specific postures, regulated breathing and meditation.[32] Yoga texts mention that the root cause of many diseases can be traced to lifestyle and amplified likes and dislikes at the mind level. The distressful emotional surges (called *aadhi*)[33] may percolate into the physical frame manifesting as diseases.[24] Hence, yoga is fast advancing as an effective therapeutic tool in physical, psychological and psychosomatic disorders.[34] In a study by Vempati *et al*. on healthy adults, the yoga-based guided relaxation was shown to reduce the sympathetic activity as measured by autonomic parameters, oxygen consumption and breath volume.[21] Medical and pre-medical students showed lesser anxiety and stress during an examination period after 8 weeks of meditation.[35] Transcendental meditation (TM) was compared to muscle relaxation in its effectiveness in controlling stress with significantly better reduction in blood pressure in the TM group.

Brain imaging studies have shown that meditation shifts the brain activity in the prefrontal cortex from the right hemisphere to the left indicating that the brain is re-oriented from a stressful fight or flight mode to one of acceptance, a shift that may indicate better contentment.[29]

Thus, the etiology of CNP being multifactorial, there is sufficient evidence in the literature to demonstrate a requirement to draw treatment options from many sources in order to achieve a favorable pain relief outcome.

The RCT design demonstrated several methodological strengths: (a) CNP of both the categories, physical (cervical spondylosis) and psychological (muscle spasm) were included in the study; (b) it used a standardized randomization procedure; (c) there was baseline matching of confounding factors such as age, sex, height, weight and BMI; (d) assessment was multidimensional including both objective and subjective parameters; (e) because the duration of the yoga intervention was short, the acceptability and adherence to the therapy was good; (f) As MSRT was played using a cassette in the therapy sessions, it could be reproduced in the exact way for all cases.

## CONCLUSION

This randomized control study has shown that yoga relaxation through MSRT adds significant complimentary benefits to conventional physiotherapy for CNP by reducing pain, disability and state anxiety and improving flexibility.

### Limitations of the study

This was a study from one orthopedic unit in Bengaluru city only. The MSRT technique used involved chanting of Indian mantra which may be unacceptable and difficult for non-Indian community. Follow up of these cases are required for compliance and recurrences.

### Suggestions for Future Work

Future studies should be done in other study groups from different orthopedic centers in India and other countries to establish the generalizability. In addition, there is a need for clinical studies to determine whether yoga-based relaxation technique can decrease medication requirement. Basic physiological studies to understand the mechanisms responsible for therapeutic effects of MSRT on CNP may be undertaken.

### Implications and recommendations

An integrative holistic model incorporating psychological and physical therapies for CNP will strengthen the rationalistic approach to treatment of CNP. We recommend that this simple procedure of using relaxation during and after the physiotherapy may be incorporated in all conventional therapy units round the globe in the management of CNP.
